# Cerebrospinal fluid analysis and changes over time in patients with subarachnoid hemorrhage: a prospective observational study

**DOI:** 10.1186/s44158-025-00250-1

**Published:** 2025-06-12

**Authors:** Alessandro Pesaresi, Denise Battaglini, Pasquale Anania, Silvia Sgambetterra, Camilla Origlia, Gianluigi Zona, Thomas Langer, Nicolò Antonino Patroniti, Pietro Fiaschi, Chiara Robba

**Affiliations:** 1 Division of Neurosurgery, IRCCS Ospedale Policlinico San Martino, Genoa, Italy; 2 Neurosurgery Unit, “Città Della Salute E Della Scienza” University Hospital, Turin, Italy; 3 Department of Neuroscience “Rita Levi Montalcini”, University of Turin, Turin, 10126 Italy; 4 Anesthesia and Critical Care, IRCCS Ospedale Policlinico San Martino, Genoa, Italy; 5 Department of Surgical Science and Integrated Diagnostics (DISC), University of Genoa, Genoa, Italy; 6 Department of Neurosciences, Rehabilitation, Ophthalmology, Genetics, Maternal and Child Health (DINOGMI), University of Genoa, Genoa, Italy; 7 Department of Medicine and Surgery, University of Milan-Bicocca, Monza, Italy; 8 Department of Anesthesia and Intensive Care Medicine, Niguarda Ca’ Granda, Milan, Italy

**Keywords:** Subarachnoid hemorrhage, Cerebrospinal fluid, Blood, Vasospasm, Hydrocephalus, Acid–base equilibrium, Electrolyte alterations, Cerebral aneurysm

## Abstract

**Background:**

Changes in cerebrospinal fluid (CSF) in patients with aneurysmal subarachnoid hemorrhage (aSAH) have not been fully elucidated, yet they are critical and may potentially be associated with the risk of complications. The aim of this study is to characterize the biochemical properties of CSF and examine the temporal changes in aSAH patients with and without post-aSAH complications such as vasospasm and shunt-dependent hydrocephalus.

**Methods:**

This prospective observational longitudinal cohort study involved collecting CSF and arterial blood samples from SAH patients requiring an external ventricular drain at four different timepoints following the initial event (1–3, 4–7, 8–13, and 14–20 days after aSAH). A control group that comprised patients with idiopathic normal pressure hydrocephalus undergoing CSF sampling was included.

**Results:**

A total of 20 SAH patients and 20 controls were enrolled. We observed significantly higher levels of hemoglobin (Hb), proteins, lactate, and cell concentrations in the CSF of aSAH patients compared to the control group (*p* < 0.001), with no corresponding differences in serum levels. Furthermore, a progressive decline in CSF Hb, proteins, and cells levels was noted over the days following the hemorrhage (*p* = 0.029, *p* = 0.005, and *p* = 0.010, respectively). Patients that developed vasospasm exhibited a lower CSF glucose/lactate ratio (*p* < 0.001) and reduced CSF sodium levels (*p* = 0.045), while patients that developed shunt-dependent hydrocephalus exhibited higher plasmatic and CSF glucose levels (*p* = 0.013 and *p* = 0.003, respectively) and lower CSF Hb/proteins ratio (*p* < 0.001).

**Conclusions:**

Patients with aSAH exhibit changes in the biochemical profile of the CSF, which evolve over time following the acute event. Parameters such as CSF glucose/lactate ratio and CSF Hb/proteins ratio could potentially provide valuable insights not only into the pathophysiology of aSAH but also into patient risks of post-hemorrhagic complications, such as vasospasm and hydrocephalus.

**Supplementary Information:**

The online version contains supplementary material available at 10.1186/s44158-025-00250-1.

## Introduction

Aneurysmal subarachnoid hemorrhage (aSAH) is a severe acute event characterized by abrupt blood extravasation into the subarachnoid spaces, typically resulting from the rupture of an aneurysm or an arterial branch [1].

Despite advances in management strategies and therapeutic protocols, aSAH remains associated with high mortality rates, ranging from 35 to 50%, and significant disability rates [2]. Moreover, the development of vasospasm and hydrocephalus further exacerbates the initial damage, leading to secondary brain injury which is well known to be associated with a poor outcomes [3–6]. Notably, up to 56% of survivors exhibits neurological deficits and neurocognitive impairment [7].

To better understand the mechanisms underlying the pathological changes that occur in the brain following subarachnoid hemorrhage, several studies have investigated the biochemical effects of free blood in cerebrospinal fluid (CSF). Froman and Crampton Smith (1966 and 1967) firstly demonstrated an increase in CSF lactic acid concentration and a decrease in CSF pH following subarachnoid hemorrhage, suggesting that the hyperventilation observed in some patients was likely due to CSF acidosis [8, 9]. In addition, the pathophysiology of acid–base equilibrium of CSF is not well elucidated and very peculiar. Indeed, CSF is generally characterized by a very low concentration of proteins, thus making the regulation of acid–base equilibrium mainly dependent on the partial pressure of carbon dioxide (PCO_2_) and the strong ion difference (SID) [10]. However, little is known about these changes after aSAH, as well as their behavior and possible role in important complications occurring in these patients, such as vasospasm or hydrocephalus. A more comprehensive understanding of the biochemical changes in CSF induced by aSAH could therefore provide valuable insights into the underlying pathophysiological mechanisms that contribute to the damage and complications associated with aSAH [11].

The primary objective of this study is to characterize the biochemical characteristics of the CSF of aSAH patients **—** comparing them with control group, as well as the temporal evolution of these changes after aSAH and their relationship with contemporary samples of arterial blood gas. Additionally, we aim to identify differences in biochemical profiles between aSAH patients who develop vasospasm or shunt dependency and those who do not.

## Methods

The current study was designed as a single-center, prospective observational longitudinal cohort study including consecutive patients with a diagnosis of aSAH who underwent external ventricular drainage (EVD) positioning at IRCCS San Martino Hospital, Genova, Italy, in the period from 1st January 2020 to 1st June 2023. This study was approved by the local ethics review board (Comitato Etico Regione Liguria, protocol no. CER Liguria: 139/2020 — DB ID 10451) and was conducted according to the “Strengthening the Reporting of Observational Studies in Epidemiology” (STROBE) statement guidelines for observational cohort studies [12] (see Additional File 1).

Inclusion criteria for the aSAH group were as follows: (1) age ≥ 18 years old, (2) radiological and clinical diagnosis of aSAH, (3) the presence of EVD for clinical purpose, and (4) availability of arterial blood and CSF gases sample for clinical purpose.

Exclusion criteria were pregnancy and the absence of informed consent.

General management of patients with aSAH followed the most recent guidelines [13].

The EVD was placed in patients with aSAH who developed acute hydrocephalus, in accordance with guidelines for the management of aSAH [14, 15]. The right frontal cerebral hemisphere was chosen as the preferred entry site for the EVD due to its non-dominance for language function in over 90% of patients. The patient was positioned supine with the head elevated at 45°. After removing hair with clippers and disinfecting the scalp, local anesthesia was administered, followed by a linear skin incision down to the bone. The periosteum was then scraped, and a burr hole was drilled at Kocher’s point to avoid the superior sagittal sinus and the motor strip of the frontal cortex. Following the opening of the dura, corticectomy was performed using bipolar forceps. The ventricular catheter was inserted up to 7 cm, targeting the medial contralateral canthus in the coronal plane and the ipsilateral tragus in the sagittal plane. Once CSF flow was observed, the catheter stylet was removed, and the proximal portion of the catheter was tunneled through the skin, away from the entry point through a separate incision. This was then sutured securely in place and connected to an external drainage system.

Another 20 patients with suspected idiopathic normal pressure hydrocephalus (iNPH) requiring a 24-h long external lumbar drainage (ELD) were selected during the same period as the control group. A lumbar puncture was performed using sterile technique usually in the lumbar 4–5 interspinous space, with a large-bore Tuohy needle (14–16 gauge). After CSF spillage was confirmed, the stylet was removed, and a 17–18-G catheter was slowly introduced into the subarachnoid space for about 10 cm. Then the needle was withdrawn, and the drain was connected to an external CSF collection system. A loop was created in the catheter to relieve tension, and it was then secured over the patient’s flank with sterile dressings. An average drainage of 10 ml of CSF per hour was accomplished to avoid over-drainage. After 24 h from ELD placement, the system was removed, and patients were regularly discharged. We excluded any samples visibly contaminated with blood.

### Samples collection

Patients diagnosed with aSAH were enrolled at the time of EVD placement (day 0). Each sample was collected between 1 and 3 days after EVD placement (first sample), between 4 and 7 days (second sample), between 8 and 13 days (third sample), and between 14 and 20 days (fourth sample). According to local protocols and clinical practice, these samples are currently performed considering the above timeframes for monitoring biochemical changes and raise the suspect of complications in these patients.

The sampling method took place according to the most up-to-date international recommendations on disinfection and sterilization procedures. Cerebrospinal fluid samples were obtained through the proximal port (closest to the head) of the EVD collecting system. Of note, the sampling was performed using strict sterile technique to minimize the risk of infection. Aspiration proceeded very slow (no more than 1 ml/min) until 7 ml of CSF was collected. The system was disinfected before and after each sampling using 0.5% alcoholic chlorhexidine. Then, 2 ml of CSF was analyzed using the same full-automatic blood gas analyzer, while the remaining 5 ml was analyzed by IRCCS San Martino Hospital laboratory to detect the presence of cells, proteins, and magnesium levels in CSF.

As for clinical practice, arterial blood gas analysis was also performed; 2 ml of whole blood was collected via radial artery puncture after disinfection. After extraction of blood, the artery was compressed using the sterile gauze for at least 5 min. At the same time, the blood was blended evenly using the artery hemostix and analyzed with the blood gas analyzer (Model: ABL90 Flex Plus). The arterial blood gas obtained for clinical reasons closest to the CSF sample was considered for pairing.

Bicarbonate radical (HCO_3_^−^), hemoglobin (Hb), lactates (Lct), glucose (Glu), sodium (Na^+^), potassium (K^+^), and chloride (Cl^−^) were measured in strict accordance with instructions of the blood gas analyzer and kit.

Moreover, the differences (Δ[]) and the ratios (Ra[]) between CSF values and arterial plasma (Pl) values were calculated as previously described [10, 11]:

- $$\bullet\;\triangle[]=[]_{CSF}-[]_{Pl}$$
- $$\bullet\;\mathrm{Ra}[]=[]_{CSF}/[]_{Pl}$$

In addition, further parameters were calculated, such as the strong ion difference (*SID* = [Na^+^] + [K^+^] + 2 × [Ca2^+^] + 2 × [Mg2^+^] − [Cl^−^] − [Lct]), the CSF Hb/proteins ratio, and the CSF glucose/lactate ratio (Glu/Lct ratio).

Data regarding aSAH etiology and classification, the onset of vasospasm and the subsequent need of surgical or endovascular treatment, and the subsequent need of ventricular-peritoneal shunt (VPS) were also collected.

Blood and CSF samples of control group were collected at the time of ELD positioning, and they were analyzed in the same way of samples of research group, as previously described.

### Vasospasm evaluation

Daily transcranial Doppler (TCD) monitoring was conducted. Patients exhibiting a mean flow velocity increase of over 50 cm/s compared to the opposite side, a velocity exceeding 120 cm/s, and with a Lindegaard ratio above 3 on TCD were referred for CT angiography (CTA) to assess for vasospasm [16–18]. When vasospasm was confirmed on CTA, endovascular intervention was carried out. If CTA findings were negative but clinical suspicion remained strong, diagnostic angiography was performed, allowing for immediate treatment in the event vasospasm was detected.

### Statistical analysis

Descriptive statistics were reported with mean and standard deviation for cardinal variables and with frequency and percentage for categorical variables. We performed the Shapiro–Wilk test to test the assumption of data normality. We performed Levene’s test to assess the equality of variances. Between-group differences were evaluated using the Mann–Whitney *U-* or independent *T*-tests, depending on the assumptions met. Differences between different sample timings were evaluated using the one-way ANOVA parametric or Kruskal–Wallis tests, depending on the assumptions met. A *p*-value < 0.05 was considered statistically significant. Statistical analysis was performed using SPSS software (version 25.0) and jamovi software (the jamovi project [2019], jamovi [version 2.3.26.0] [computer software], https://www.jamovi.org).

## Results

### Population study

Twenty consecutive patients with a diagnosis of aSAH were prospectively enrolled, comprising 10 males (50%) and 10 females (50%). The mean age (standard deviation, SD) was 61 ± 10 years, ranging from 43 to 74 years. Only three patients were intubated at the time of admission (15%). No patients received hypertonic saline or mannitol.

In 6 cases, the aneurysm was in the posterior circulation (30%), while in 14 cases the anterior circulation was involved (70%); aneurysmal rupture was treated endovascularly in 16 cases (80%, with 14 cases involving coiling and 2 cases involving stent placement), while 4 underwent surgical clipping (16%). During hospitalization, three patients developed vasospasm (15%), and nine patients required VPS placement (45%). Vasospasm occurred between days 8–13 in one patient and between days 14–20 in two cases. One patient died during hospitalization (5%).

The control group consisted of 20 consecutive patients (11 males, 55%; and nine females, 45%) with suspected iNPH who underwent ELD with a mean age of 66 ± 9 years, ranging from 48 to 81 years. Clinical data are summarized in Additional File 2.

### Comparison between research and control group

Cerebrospinal fluid of patients with aSAH had higher concentration of hemoglobin (0.87 ± 1.05 g/dL vs. 0.12 ± 0.29 g/dL, *p* < 0.001) and lactate (3.2 ± 0.8 mmol/L vs. 1.9 ± 0.5 mmol/L, *p* < 0.001), while potassium concentration was lower (2.28 ± 0.30 mmol/L vs. 2.38 ± 0.19 mmol/L, *p* = 0.013) as compared to the control group. Patients with aSAH also showed higher cell count (104.0 ± 99.0 n/mmc vs. 33.9 ± 116 n/mmc, *p* < 0.001) and higher concentrations of proteins (11.1 ± 9.90 mg/dL vs. 4.72 ± 3.18 mg/dL, *p* < 0.001) than controls.

When considering the differences between CSF and arterial blood samples, patients with aSAH showed higher values of *Δ*Lct (2.31 ± 0.85 mmol/L vs. 0.08 ± 0.87 mmol/L, *p* < 0.001) but lower values of *Δ*Glu (− 57.98 ± 23.94 mg/dL vs. − 45.65 ± 33.00 mg/dL, *p* = 0.010).

Finally, analyzing the ratio between CSF and blood values, patients with aSAH exhibited lower values of RaHb (0.01 ± 0.02 vs. 0.06 ± 0.08, *p* < 0.001). No statistically significant differences were observed regarding the other indices considered (Table 1).

**Table 1:** Comparison between research and control group

	Blood			CSF			*Δ* (CSF-blood)			Ra (CSF/blood)		
Detected indexes	Research group	Control group	*p*-value	Research group	Control group	*p*-value	Research group	Control group	*p*-value	Research group	Control group	*p*-value
Hb (g/dL)	13.6 ± 3.48	12 ± 2.09	0.086*	0.87 ± 1.05	0.12 ± 0.29	< 0.001*	− 12.64 ± 0.49	− 11.92 ± 2.09	0.682*	0.01 ± 0.02	0.06 ± 0.08	< 0.001*
Glu (mg/dL)	129 ± 33.3	113 ± 38.3	0.008*	72 ± 21.8	67.6 ± 11.3	0.869*	− 57.98 ± 23.94	− 45.65 ± 33.00	0.010*	0.59 ± 0.63	0.55 ± 0.14	0.125*
Lct (mmol/L)	0.93 ± 0.35	1.09 ± 0.85	0.430*	3.24 ± 0.77	1.90 ± 0.51	< 0.001*	2.31 ± 0.85	0.08 ± 0.87	** < 0.001^**	2.38 ± 1.29	3.97 ± 1.72	< 0.001*
Na^+^ (mmol/L)	140 ± 6.92	139 ± 3.37	0.639*	143 ± 5.65	141 ± 5.50	0.219*	3.31 ± 4.10	2.4 ± 5.26	0.233*	1.01 ± 0.04	1.02 ± 0.03	0.258*
K^+^ (mmol/L)	3.59 ± 0.42	3.69 ± 0.31	0.361^	2.28 ± 0.30	2.38 ± 0.19	0.013*	− 1.31 ± 0.52	− 1.30 ± 0.60	0.681*	0.62 ± 0.15	0.63 ± 0.17	0.451*
Cl^−^ (mmol/L)	107 ± 7.14	107 ± 4.74	0.721*	119 ± 4.49	117 ± 4.82	0.334^	13.21 ± 5.37	10.75 ± 5.65	0.080*	1.10 ± 0.05	1.12 ± 0.06	0.172*
HCO3^−^ (mmol/L)	25.2 ± 3.31	25.3 ± 2.18	0.845*	21.7 ± 2.37	22.3 ± 1.67	0.405^	− 3.84 ± 2.94	3.06 ± 2.57	0.228*	0.88 ± 0.10	0.86 ± 0.11	0.190*
SID	36.11 ± 4.20	34.89 ± 2.87	0.241^	22.69 ± 3.72	24.14 ± 8.39	0.463*	− 13.74 ± 5.80	− 10.48 ± 8.39	0.069*	0.64 ± 0.14	0.65 ± 0.16	0.199*
Cells (n/mmc)	-	-		104.0 ± 99.0	33.9 ± 116	< 0.001*	-	-		-	-	
Proteins (mg/dL)	-	-		11.1 ± 9.90	4.72 ± 3.18	< 0.001*	-	-		-	-	

*Cl* chloride, *CSF* cerebrospinal fluid, *Glu* glucose, *Hb* hemoglobin, *HCO*_*3*_^*−*^ bicarbonate radical, *K*^+^ potassium, *Lct* lactate, *Na*^+^ sodium, *SID* strong ion difference

^*^Mann–Whitney U-test. ^Independent t-test. Statistically significative values were reported in bold

### Comparison of different timing of sample collection in aSAH patients

**Fig. 1:**
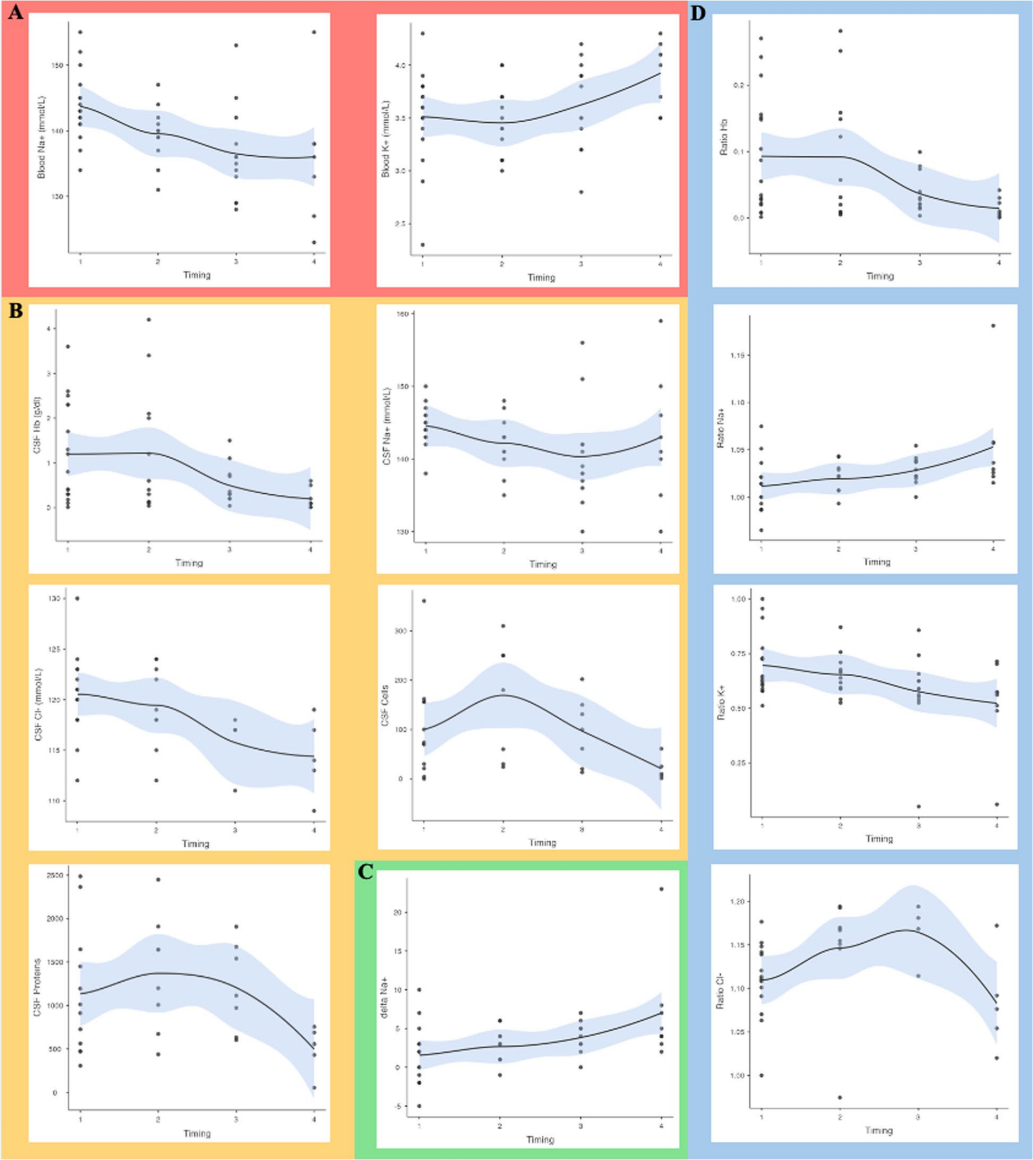
Graphical representation of the variation over the different considered time points of the analyzed parameters in aSAH patients across the various samples. **A** Blood samples. **B** CSF samples. **C** Delta (CSF-blood). **D** Ratio (CSF/blood). Cl, chloride; Glu, glucose; Hb, hemoglobin; K^+^, potassium; Na^+^, sodium; Prot, proteins; aSAH, aneurismal subarachnoid hemorrhage

Blood samples showed a decrease in Na^+^ levels (from 144 ± 5.21 at days 1–3 to 137 ± 9.50 at days 14–21, *p* = 0.003) and an increase in K^+^ levels (from 3.51 ± 0.45 at days 1–3 to 3.92 ± 0.32 at days 14–21, *p* = 0.027) (Fig. 1A).

Comparing samples collected at different time windows after EVD placement, we observed a progressive decrease in Cl − levels (days 1–3: 121 ± 4.09 mmol/L; days 4–7: 119 ± 4.19 mmol/L; days 8–13: 116 ± 8.20 mmol/L; days 14–21: 114 ± 3.85 mmol/L, *p* = 0.048). Regarding the concentration of hemoglobin in the CSF, we found an initial increase from days 1–3 (1.19 ± 1.1 g/dL) to days 4–7 (1.22 ± 1.41 g/dL), followed by a progressive decrease (days 8–13: 0.50 ± 0.44 g/dL; days 14–21: 0.20 ± 0.23 g/dL, *p* = 0.029). We also observed statistically significant differences in sodium levels, which peaked on days 1–3 (145 ± 2.86 mmol/L) and then gradually decreased on days 4–7 and days 8–13 (142 ± 3.74 mmol/L and 140 ± 7.10 mmol/L, respectively), before showing a subsequent increase on days 13–21 (143 ± 8.94 mmol/L). Interestingly, this trend over time was also present for other variables considered (Glu, Lct, K^+^, HCO3^−^), although without statistically significant differences. On contrary, cells and proteins showed an increase in days 4–7 and then a progressive decrease (*p* = 0.029, *p* = 0.005, and *p* = 0.010, respectively) (Fig. 1B).

Additionally, a progressive reduction in serum Na^+^ levels was noted (*p* = 0.003), with the maximum *Δ*Na^+^ observed on days 13–21 (7.00 ± 6.76 mmol/L), while days 4–7 showed higher Na^+^ levels in the blood compared to the CSF (*Δ*Na^+^ : − 2.67 ± 2.15 mmol/L) (*p* = 0.018) (Fig. 1C).

Finally, analyzing the ratio between CSF and blood values, we observed a progressive decrease in RaHb (days 1–3: 1.02 ± 0.02; days 4–7: 0.90 ± 0.32; days 8–13: 0.84 ± 0.40; days 14–21: 0.85 ± 0.42, *p* = 0.037), RaHb (days 1–3: 0.09 ± 0.09; days 4–7: 0.09 ± 0.10; days 8–13: 0.04 ± 0.03; days 14–21: 0.01 ± 0.02, *p* = 0.037), and RaK^+^ (days 1–3: 0.70 ± 0.14; days 4–7: 0.65 ± 0.10; days 8–13: 0.58 ± 0.19; days 14–21: 0.52 ± 0.20, *p* = 0.045). Furthermore, statistically significant differences were also observed for and RaCl − (*p* = 0.013) (Fig. 1D).

All data are reported in Table 2.

**Table 2:** Analysis of different timing of sample collection in aSAH patients

Detected indexes	Days 1–3	Days 4–7	Days 8–13	Days 14–20	*p*-value
**Blood indexes**					
Hb (g/dL)	13.4 ± 3.33	13.5 ± 3.56	13.6 ± 2.77	14.3 ± 4.99	0.979*
Glu (mg/dl)	130 ± 33.3	128 ± 30.2	131 ± 35.9	130 ± 40.7	0.984*
Lct (mmol/L)	0.98 ± 0.40	0.88 ± 0.31	0.96 ± 0.38	0.89 ± 0.24	0.933*
Na^+^ (mmol/L)	144 ± 5.21	140 ± 4.03	136 ± 7.28	137 ± 9.50	0.003*
K^+^ (mmol/L)	3.51 ± 0.45	3.45 ± 0.34	3.63 ± 0.43	3.92 ± 0.32	**0.027^**
Cl^−^ (mmol/L)	109 ± 4.36	106 ± 6.42	104 ± 9.05	105 ± 9.51	0.125*
HCO3^−^ (mmol/L)	24.8 ± 2.03	26.0 ± 3.99	24.3 ± 3.76	26.1 ± 3.87	0.058^
SID	37.15 ± 3.69	36.68 ± 4.42	35.46 ± 3.86	33.91 ± 5.07	0.403^
**CSF indexes**					
Hb (g/dL)	1.19 ± 1.11	1.22 ± 1.41	0.50 ± 0.44	0.20 ± 0.23	0.029*
Glu (mg/dl)	75 ± 18.2	70 ± 21.2	65 ± 24.0	79 ± 26.9	0.363*
Lct (mmol/L)	3.19 ± 0.80	3.17 ± 0.78	3.52 ± 0.80	3.85 ± 0.69	0.553^
Na^+^ (mmol/L)	145 ± 2.86	142 ± 3.74	140 ± 7.10	143 ± 8.94	0.040*
K^+^ (mmol/L)	2.41 ± 0.38	2.18 ± 0.20	2.19 ± 0.19	2.31 ± 0.34	0.085*
Cl^−^ (mmol/L)	121 ± 4.09	119 ± 4.19	116 ± 8.20	114 ± 3.85	**0.048^**
HCO3 − (mmol/L)	21.8 ± 2.47	21.1 ± 2.03	21.1 ± 3.04	23.2 ± 2.05	0.402^
SID	22.77 ± 4.06	21.71 ± 3.05	21.55 ± 2.97	25.12 ± 4.11	0.471^
Cells (n/mmc)	100.3 ± 100.4	169.3 ± 114.6	96.7 ± 69.9	20.6 ± 24.3	**0.005^**
Proteins (mg/dL)	11.3 ± 7.3	13.7 ± 6.7	12.1 ± 5.2	4.9 ± 2.7	**0.010^**
*Δ* **(CSF-blood)**					
Hb (g/dL)	− 12.24 ± 3.71	− 11.75 ± 2.40	− 13.13 ± 12.95	− 14.05 ± 4.92	0.590*
Glu (mg/dl)	− 54.82 ± 20.32	− 59.75 ± 27.36	− 65.42 ± 26.40	− 50.88 ± 23.08	0.522*
Lct (mmol/L)	2.20 ± 0.95	2.31 ± 0.84	2.56 ± 0.84	2.18 ± 0.78	0.704^
Na + (mmol/L)	1.56 ± 3.76	− 2.67 ± 2.15	3.83 ± 1.90	7.00 ± 6.76	0.018*
K + (mmol/L)	− 1.09 ± 0.58	− 1.27 ± 0.42	− 1.43 ± 0.48	− 1.65 ± 0.14	0.067^
Cl − (mmol/L)	11.80 ± 4.46	15.00 ± 7.00	16.25 ± 2.99	11.80 ± 5.50	0.055*
HCO3 − (mmol/L)	− 2.82 ± 3.04	− 5.85 ± 1.40	− 4.10 ± 2.30	− 3.22 ± 3.87	0.069^
SID	− 13.29 ± 5.53	− 15.84 ± 5.65	− 15.80 ± 4.34	− 9.69 ± 6.98	0.194*
**Ra (CSF/blood)**					
Hb (g/dL)	0.09 ± 0.09	0.09 ± 0.10	0.04 ± 0.03	0.01 ± 0.02	0.046*
Glu (mg/dl)	0.56 ± 0.15	0.55 ± 0.14	0.50 ± 0.13	0.61 ± 0.12	0.345*
Lct (mmol/L)	3.91 ± 1.92	4.17 ± 1.96	4.04 ± 1.58	3.66 ± 1.31	0.932*
Na^+^ (mmol/L)	1.01 ± 0.03	1.02 ± 0.02	1.03 ± 0.01	1.05 ± 0.05	0.010*
K^+^ (mmol/L)	0.70 ± 0.14	0.65 ± 0.10	0.58 ± 0.19	0.52 ± 0.20	0.045*
Cl^−^ (mmol/L)	1.11 ± 0.04	1.15 ± 0.07	1.16 ± 0.04	1.08 ± 0.06	0.013*
HCO3^−^ (mmol/L)	0.89 ± 0.12	0.78 ± 0.05	0.84 ± 0.08	0.89 ± 0.13	0.077*
SID	0.64 ± 0.14	0.59 ± 0.13	0.59 ± 0.08	0.73 ± 0.17	0.152*

*Cl* chloride, *Glu* glucose, *Hb* hemoglobin, *HCO*_*3*_^*−*^ bicarbonate radical, *K*^+^ potassium*, Lct* lactate, *Na*^+^ sodium, *SID* strong ion difference, *aSAH* aneurysmal subarachnoid hemorrhage

^*^Kruskal–Wallis test. ^One-way ANOVA test. Statistically significative values were reported in bold

### Differences between patients who developed vasospasm and not in aSAH patients

Patients who developed vasospasm, compared to those who did not, had lower levels of sodium in CSF (136.0 ± 8.8 vs. 143.0 ± 5.0, *p* = 0.045). Full analysis is reported in Table 3.

**Table 3:** Analysis of blood and cerebrospinal fluid factors in patients who developed — vs who did not — vasospasm in aSAH patients

	Blood			CSF			*Δ* (CSF-blood)			Ra (CSF/blood)		
Detected indexes	Vasospasm group	No vasospasm group	*p*-value	Vasospasm grou**p**	No vasospasm group	*p*-value	Vasospasm group	No vasospasm group	*p*-value	Vasospasm group	No vasospasm group	*p*-value
Hb (g/dL)	12.7 ± 2.1	13.8 ± 4.2	0.368*	0.35 ± 0.43	0.36 ± 1.07	0.797*	− 11.9 ± 2.3	− 12.7 ± 4.5	0.655*	0.0 ± 0.0	0.0 ± 0.1	0.808*
Glu (mg/dL)	125.0 ± 33.0	121.0 ± 36.5	0.917*	63.5 ± 16.0	68.0 ± 35.0	0.074*	− 65.5 ± 43.8	− 53.0 ± 26.0	0.329^	0.5 ± 0.2	0.6 ± 0.1	0.317*
Lct (mmol/L)	0.90 ± 0.93	0.85 ± 0.40	0.358*	3.1 ± 1.1	3.2 ± 1.0	0.803*	1.8 ± 1.3	2.3 ± 1.2	0.347^	3.0 ± 2.3	3.7 ± 2.2	0.268*
Na^+^ (mmol/L)	134.0 ± 11.5	140.0 ± 6.0	0.156*	136.0 ± 8.8	143.0 ± 5.0	0.045*	2.0 ± 1.8	3.0 ± 4.0	0.225*	1.0 ± 0.0	1.0 ± 0.0	0.391*
K^+^ (mmol/L)	3.65 ± 0.68	3.60 ± 0.48	0.604*	2.2 ± 0.5	2.2 ± 0.2	0.731*	− 1.3 ± 0.6	− 1.4 ± 0.7	0.903^	0.6 ± 0.1	0.6 ± 0.1	0.860*
Cl^−^ (mmol/L)	102.0 ± 9.5	106.0 ± 9.0	0.193*	121.0 ± 5.3	118.0 ± 5.0	0.543*	14.0 ± 2.8	15.0 ± 7.0	0.803*	1.1 ± 0.0	1.1 ± 0.1	1.000*
HCO3^-^ (mmol/L)	25.2 ± 1.9	25.4 ± 3.5	0.695*	22.6 ± 2.7	21.5 ± 3.7	0.930*	− 3.2 ± 2.1	− 4.3 ± 3.4	0.536*	0.9 ± 0.1	0.8 ± 0.1	0.629*
SID	36.15 ± 3.3	36.10 ± 4.3	0.873*	21.0 ± 2.0	22.5 ± 3.8	0.720*	− 16.1 ± 3.2	− 13.4 ± 6.0	0.363*	0.6 ± 0.1	0.6 ± 0.2	0.540*
Cells (n/mmc)	-	-		250.0 ± 174.0	70.0 ± 132.0	0.271*	-	-		-	-	
Proteins (mg/dL)	-	-		10.1 ± 3.4	9.1 ± 10.8	0.583*	-	-		-	-	

Data are reported as median ± interquartile range (IQR)

*Cl* chloride, *CSF* cerebrospinal fluid, *Glu* glucose, *Hb* hemoglobin, *HCO*_*3*_^*−*^ bicarbonate radical, *K*^+^ potassium, *Lct* lactate, *Na*^+^ sodium, *SID* strong ion difference, *aSAH* aneurysmal subarachnoid hemorrhage

^*^Mann–Whitney *U*-test. Statistically significative values were reported in bold

### Differences between patients who required VPS dependency in aSAH patients

In patients who underwent VPS placement after aSAH, we observed higher glucose levels in CSF (81.92 ± 23.69 mg/dL vs. 62.44 ± 14.73 mg/dL, *p* = 0.003) and higher HCO3^−^ levels (22.64 ± 2.74 mmol/L vs. 20.90 ± 1.64 mmol/L, *p* = 0.039). Complete analysis is reported in Table 4.

**Table 4:** Analysis of blood and cerebrospinal fluid factors in patients requiring VPS in aSAH patients

	Blood			CSF			*Δ* (CSF-blood)			Ra (CSF/blood)		
Detected indexes	VPS group	No VPS group	*p*-value	VPS group	No VPS group	*p*-value	VPS group	No VPS group	*p*-value	VPS group	No VPS group	*p*-value
Hb (g/dL)	14.11 ± 3.76	13.11 ± 3.17	0.292*	0.72 ± 0.95	1.02 ± 1.14	0.316*	− 13.02 ± 3.41	− 12.23 ± 3.46	0.303*	0.05 ± 0.07	0.08 ± 0.09	0.230*
Glu (mg/dL)	142.24 ± 38.48	117.15 ± 21.73	0.013*	81.92 ± 23.69	62.44 ± 14.73	0.003*	− 60.24 ± 20.84	− 55.83 ± 26.95	0.419^	0.58 ± 0.15	0.51 ± 0.12	0.113^
Lct (mmol/L)	0.93 ± 0.42	0.94 ± 0.25	0.428*	3.10 ± 0.77	3.38 ± 0.76	0.207^	2.17 ± 0.86	2.46 ± 0.84	0.241^	4.01 ± 2.04	3.92 ± 1.36	0.741*
Na + (mmol/L)	139.46 ± 6.17	139.88 ± 7.79	0.835	141.38 ± 4.61	143.92 ± 6.38	0.121^	2.58 ± 2.62	4.04 ± 5.13	0.298*	1.02 ± 0.02	1.03 ± 0.04	0.433*
K + (mmol/L)	3.59 ± 0.47	3.59 ± 0.35	0.968^	2.25 ± 0.23	2.32 ± 0.37	0.640*	− 1.35 ± 0.50	− 1.27 ± 0.55	0.588^	0.60 ± 0.19	0.66 ± 0.13	0.373*
Cl − (mmol/L)	105.12 ± 5.99	108.17 ± 8.04	0.140^	118.47 ± 3.57	119 ± 5.42	0.741^	13.82 ± 3.91	12.56 ± 6.66	0.986*	1.12 ± 0.05	1.12 ± 0.07	0.719*
HCO3 − (mmol/L)	25.2 ± 4.32	25.22 ± 1.91	0.831*	22.64 ± 2.74	20.90 ± 1.64	**0.039^**	− 3.27 ± 3.59	− 4.37 ± 3.59	0.304^	0.88 ± 0.13	0.82 ± 0.08	0.281*
SID	37.26 ± 4.54	34.84 ± 3.45	**0.043^**	23.22 ± 2.45	24.13 ± 4.85	0.052*	− 14.36 ± 5.10	− 13.09 ± 6.57	0.829*	0.63 ± 0.11	0.64 ± 0.17	0.423*
Cells (n/mmc)	-	-		124.0 ± 114.0	82.5 ± 77.3	0.416^	-	-		-	-	
Proteins (mg/dL)	-	-		12.30 ± 6.49	9.61 ± 6.66	0.271*	-	-		-	-	

*Cl* chloride, *CSF* cerebrospinal fluid, *Glu* glucose, *Hb* hemoglobin, *HCO*_*3*_^−^ bicarbonate radical, *K*^+^ potassium, *Lct* lactate, *Na*^+^ sodium, *SID* strong ion difference, *aSAH* aneurysmal subarachnoid hemorrhage

^*^Mann–Whitney U-test. ^Independent t-test. Statistically significative values were reported in bold

### Specific analysis of CSF Hb/proteins ratio and Glu/Lct ratio

Although no statistically significant differences were observed regarding the concentration of hemoglobin and proteins in the CSF, the Hb/proteins ratio was lower in patients who underwent VPS (0.05 ± 0.06) compared to those with aSAH who did not require VPS (0.28 ± 0.26) (*p* < 0.001). Moreover, these differences were observed in each considered timepoint.

**Fig. 2:**
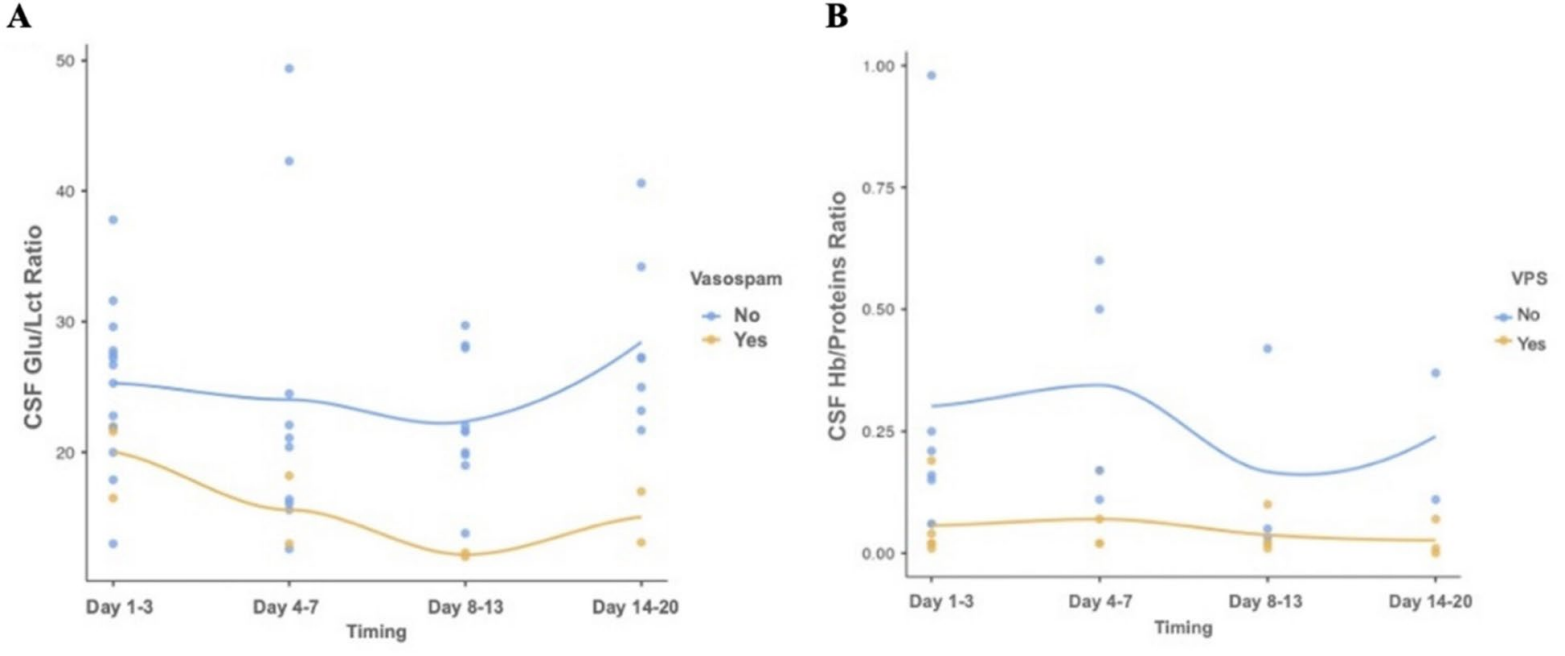
Analysis of CSF Hb/proteins ratio and CSF Glu/Lct ratio in aSAH patients during the period after hemorrhagic event. **A** Temporal trend of CSF Hb/proteins ratio after aSAH event in patients with and without hydrocephalus requiring VPS. **B** Temporal trend of CSF Glu/Lct ratio after aSAH event in patients with and without vasospasm. CSF, cerebrospinal fluid; Glu, glucose; Hb, hemoglobin; Lct, lactate; aSAH, aneurismal subarachnoid hemorrhage; VPS, ventricular-peritoneal shunt

Furthermore, patients with aSAH had a higher Hb/proteins ratio compared to the control group (0.16 ± 0.21 vs. 0.02 ± 0.03, *p* < 0.001), while no statistically significant differences were observed between the group that developed vasospasm and other aSAH patients (Fig. 2A).

Considering the CSF Glu/Lct ratio, the research group displayed significantly lower mean values compared to the control group (23.22 ± 8.07 vs. 37.92 ± 10.50, *p* < 0.001).

Regarding complication risk, patients who developed vasospasm had a lower CSF Glu/Lct ratio (16.21 ± 3.88 vs. 24.83 ± 7.94, *p* < 0.001). Lower values were also observed in patients who underwent VPS, although this association was not statistically significant (*p* > 0.05) (Fig. 2B).

## Discussion

In this study, we observed that patients with aSAH exhibit biochemical characteristics of the CSF that differ from those of control group. Moreover, among patients with aSAH, we identified specific alterations in the CSF associated with the onset of post-aSAH complications, such as hydrocephalus or vasospasm. These values also change over time following the acute event.

To our knowledge, this is the first study to assess how these indices evolve over time after the hemorrhagic event and whether they correlate with post-aSAH complications, particularly in relation to arterial values.

CSF changes in patients with aSAH have been only partially understood, but are of great interest, as they reflect the nervous system’s response to the detrimental effects of the hemorrhage and may potentially be related to the risk of complications such as vasospasm, hydrocephalus, and metabolic disturbances [19, 20].

aSAH causes the rupture of blood vessels in the central nervous system, leading to the release of red blood cells into the CSF. In our study, we observed significantly higher levels of hemoglobin in the CSF of patients with aSAH compared to the control group (*p* < 0.001), with no corresponding differences in serum Hb levels. Furthermore, we found a progressive reduction in Hb levels over the days following the hemorrhage, indicating gradual reabsorption of the subarachnoid blood component (*p* = 0.029). The presence of free hemoglobin in the CSF serves as an early indicator of hemorrhage and can act as a marker of brain injury and inflammation, and it has been associated with worse outcomes in aSAH patients [21–24].

We also observed significantly elevated protein levels and cell concentrations in the CSF of aSAH patients (*p*< 0.001). Increases in both CSF protein levels and cell count are considered biomarkers of inflammation and disruption of the blood–brain barrier (BBB) in a range of neurological diseases, including aSAH: these changes have been identified as potential mechanisms of brain injury following aSAH [25]. Moreover, as time passed from the acute event, we noted a decrease in both protein levels (*p* = 0.005) and cell count in the CSF (*p* = 0.010), which likely reflects a progressive reduction in the inflammatory response and a gradual restoration of BBB integrity.

Patients with aSAH had higher lactate levels in the CSF compared to controls (*p* < 0.001). Langer et al. reported that lactate levels in SAH patients were elevated compared to a population of healthy controls [10]. Several studies have shown that increased lactate levels in the CSF of aSAH patients are associated with poorer outcomes [26–29]. Lactate is produced in the central nervous system (CNS) through aerobic glycolysis by astrocytes, which then transfer it to neurons to serve as an alternative energy source when glucose is limited during cerebral injury. Structural changes in the endothelium during vasospasm are believed to be linked to a failure in cellular energy metabolism, resulting in a shift to anaerobic glycolysis, glycogen breakdown, and the subsequent accumulation of lactate in brain tissue as a byproduct of vasospasm [30].

Although our results did not show a statistically significant difference between CSF lactate levels and the development of post-aSAH complications, patients who developed vasospasm had a significantly lower CSF Glu/lactate ratio (*p* < 0.001). Moreover, in patients with vasospasm, the ratio was lower at all time points, with a progressive decrease observed until the interval between days 8 and 13, when the risk of vasospasm is at its highest. Taccone et al. described how SAH patients with a low CSF Glu/lactate ratio experienced worse outcomes [31]: these findings suggest that this parameter may not only serve as a prognostic marker but also as a potential predictor of vasospasm risk, despite further studies are required to confirm this.

Our data also revealed that patients with aSAH exhibited higher blood glucose levels compared to controls (*p* = 0.008). This finding has been reported in previous studies and is thought to be linked to increased stress and inflammatory responses following aSAH, which activate the hypothalamic–pituitary–adrenal axis and the sympathetic autonomic nervous system [32, 33].

It was then assessed whether patients who developed complications following aSAH (such as vasospasm or the need for a VPS) exhibited differences in the indices analyzed. In this study, patients with vasospasm had significantly lower sodium levels in the CSF (*p* = 0.026). Several studies have correlated both hyponatremia and hypernatremia with worse outcomes in patients with aSAH [34–37]. Furthermore, the current American Heart Association guidelines state that hyponatremia is associated with vasospasm and recommend the use of fludrocortisone and hypertonic saline to prevent and correct hyponatremia in patients with aSAH [2]. In a study by Melissa et al., sodium levels were associated with a higher risk of vasospasm only in samples collected after the 14 th day following the onset of aSAH [38]. Uozumi et al. demonstrated that hyponatremia in aSAH patients leads to low serum osmolarity, resulting in brain edema, which can affect the brain’s microcirculation and worsen vasospasm [39]. In our study, although patients with vasospasm had lower plasmatic sodium levels, this difference was not statistically significant. However, they exhibited lower sodium levels in the CSF (*p* = 0.045). Lower sodium levels in the CSF may undergo earlier changes than serum levels and have a greater osmotic effect on the brain, potentially increasing the risk of vasospasm. Moreover, we observed that sodium levels in patients with aSAH were lowest between the 8 th and 13 th day (the period associated with the highest risk of vasospasm) and highest between the 1 st and 3rd day (also associated with a higher risk of vasospasm), both in serum samples (*p* = 0.003) and CSF samples (*p* = 0.040).

Patients with higher glucose levels in the blood and CSF had a greater risk of developing hydrocephalus that required VPS (*p* = 0.013 and *p* = 0.003, respectively). These findings have also been described in previous studies, which suggest that VPS dependency due to hyperglycemia may be driven by several mechanisms, including inflammation, disruption of immune function, and endothelial dysfunction [40, 41].

Finally, we observed higher protein concentrations in the CSF of patients with hydrocephalus, though this difference was not statistically significant. Wilhelmy et al. documented a reduction in CSF protein levels in patients with obstructive hydrocephalus but not in those with other types of hydrocephalus: they hypothesized that in conditions leading to the accumulation of CSF in the ventricular system (such as obstructive hydrocephalus), the body initiates a compensatory mechanism by lowering protein concentrations in the CSF to facilitate reabsorption. In contrast, this compensatory mechanism may be impaired in other forms of hydrocephalus, potentially contributing to their pathogenesis (e.g., in communicating hydrocephalus). In the case of hydrocephalus following SAH, the primary cause is impaired CSF reabsorption, resulting in communicating hydrocephalus [42]. In our study, we evaluated the ratio of Hb to protein concentration in the CSF. Although the CSF Hb/proteins ratio was lower in the control group compared to the research group (*p* < 0.001), primarily due to lower Hb levels in the CSF in the control group, patients who required a VPS exhibited lower CSF Hb/protein ratios compared to those who did not develop hydrocephalus (*p* < 0.001), like the control group values. Based on these observations, we hypothesized that aSAH patients who develop hydrocephalus maintain elevated total protein levels despite a progressive decrease in Hb levels and proteins in the CSF that are observed in patients with aSAH (*p* = 0.029 and *p* = 0.010, respectively), indicating a loss of the previously described compensatory mechanisms.

### Clinical message

These findings of this study suggest that biochemical analysis of CSF in patients with aSAH may provide valuable insights into the risk of developing secondary complications such as vasospasm and hydrocephalus. Specifically, early alterations in CSF sodium levels, Glu/lactate ratio, and Hb/proteins ratio may precede clinical manifestations and radiological evidence of these complications, offering a potential window for early intervention. Monitoring these indices dynamically over time could aid in stratifying patient risk, guiding therapeutic decisions, and optimizing the timing of interventions. Incorporating CSF analysis into clinical practice may improve individualized care and potentially enhance outcomes in patients with aSAH.

### Limitations

We must acknowledge several limitations of our study. First, the relatively small sample size, particularly the limited number of patients who developed vasospasm, constrained the statistical power of our analysis. As a result, it was not possible to develop a robust prognostic model adjusted for potential covariates and confounding factors. Second, CSF samples were collected from different anatomical sites—lumbar in the control group and ventricular in aSAH patients—which may have introduced variability in the results. Third, we did not account for the type and volume of intravenous fluids administered to aSAH patients. Further studies with larger cohorts are needed to validate and expand upon our findings.

## Conclusion

Patients with aSAH exhibit changes in the biochemical profile of the CSF, which evolve over time following the acute event. Parameters such as CSF Glu/Lct ratio and CSF Hb/proteins ratio could potentially provide valuable insights not only into the pathophysiology of aSAH but also into patient risks of posthemorrhagic complications, such as vasospasm and hydrocephalus. This could enhance clinical management and pave the way for novel therapeutic strategies aimed at minimizing brain damage and improving long-term outcomes.

## Supplementary Information

Additional File 1. Supplementary Table 1. Checklist according to “Strengthening the Reporting of Observational Studies in Epidemiology” (STROBE) statement guidelines for observational cohort studies.

Additional File 2. Supplementary Table 2. Clinical data of research group. H&H: Hunt and Hess; WFNS: World Federation of Neurological Surgeons; VPS: ventricular-peritoneal shunt.

## Data Availability

Data are available after request to the PI.

## Abbreviations

aSAH: Aneurysmal subarachnoid hemorrhage
BBB: Blood-brain barrier
Cl^−^: Chloride
CNS: Central nervous system
CSF: Cerebrospinal fluid
CTA: Computed tomography angiography
ELD: External lumbar drainage
EVD: External ventricular drainage
Glu: Glucose
Hb: Hemoglobin
HCO_3_^−^: Bicarbonate radical
iNPH: Idiopathic normal pressure hydrocephalus
K^+^: Potassium
Lct: Lactates
Na^+^: Sodium
PCO2: Carbone dioxide
SID: Strong ion difference
STROBE: Strengthening the reporting of observational studies in epidemiology
TCD: Transcranial doppler
VPS: Ventricular-peritoneal shunt
