# Supplementary material for: Cerebrospinal fluid analysis and changes over time in patients with subarachnoid hemorrhage: a prospective observational study
Source: J Anesth Analg Crit Care. 2025 Jun 12;5:31. doi: 10.1186/s44158-025-00250-1 (PMC12160409; doi:10.1186/s44158-025-00250-1)
Supplement: Supplementary file 2 — Additional File 2. Supplementary Table 2. Clinical data of research group. H&H: Hunt and Hess; WFNS: World Federation of Neurological Surgeons; VPS: ventricular-peritoneal shunt. [file 44158_2025_250_MOESM2_ESM.doc]

| **Research Group Features** | **Values** |
| --- | --- |
| **Gender** |  |
| Male | 10 (50%) |
| Female | 10 (50%) |
| **Mean Age (years)** | 61.1 ± 9.54 (range: 43-74) |
| **mFisher** |  |
| Grade 1 | 1 (5%) |
| Grade 2 | 0 (0%) |
| Grade 3 | 3 (15%) |
| Grade 4 | 16 (80%) |
| **H&H Scale** |  |
| Grade 1 | 4 (20%) |
| Grade 2 | 4 (20%) |
| Grade 3 | 9 (55%) |
| Grade 4 | 1 (5%) |
| Grade 5 | 2 (10%) |
| **WFNS Scale** |  |
| Grade 1 | 8 (40%) |
| Grade 2 | 5 (25%) |
| Grade 3 | 1 (5%) |
| Grade 4 | 4 (20%) |
| Grade 5 | 2 (10%) |
| **Aneurism Location** |  |
| Anterior Circulation | 14 (70%) |
| Posterior Circulation | 6 (30%) |
| **Vasospasm** |  |
| Yes | 3 (15%) |
| No | 17 (85%) |
| **VPS** |  |
| Yes | 9 (45%) |
| No | 11 (55%) |
| **Intubated at admission** |  |
| Yes | 3/20 (15%) |
| No | 17/20 (85%) |
| **Mortality** | 1/20 (5%) |

Supplementary Table 2. Clinical data of research group. H&H: Hunt and Hess; WFNS: World Federation of Neurological Surgeons; VPS: ventricular-peritoneal shunt.
